# *Lactococcus garvieae*: Where Is It From? A First Approach to Explore the Evolutionary History of This Emerging Pathogen

**DOI:** 10.1371/journal.pone.0084796

**Published:** 2013-12-31

**Authors:** Chiara Ferrario, Giovanni Ricci, Christian Milani, Gabriele Andrea Lugli, Marco Ventura, Giovanni Eraclio, Francesca Borgo, Maria Grazia Fortina

**Affiliations:** 1 Department of Food, Environmental and Nutritional Sciences (DeFENS) - Division of Food Microbiology and Bioprocesses, Università degli Studi di Milano, Milan, Italy; 2 Department of Life Sciences, Laboratory of Probiogenomics, Università di Parma, Parma, Italy; Catalan Institute for Water Research (ICRA), Spain

## Abstract

The population structure and diversity of *Lactococcus garvieae*, an emerging pathogen of increasing clinical significance, was determined at both gene and genome level. Selected lactococcal isolates of various origins were analyzed by a multi locus sequence typing (MLST). This gene-based analysis was compared to genomic characteristics, estimated through the complete genome sequences available in database. The MLST identified two branches containing the majority of the strains and two branches bearing one strain each. One strain was particularly differentiated from the other *L. garvieae* strains, showing a significant genetic distance. The genomic characteristics, correlated to the MLST-based phylogeny, indicated that this “separated strain” appeared first and could be considered the evolutionary intermediate between *Lactococcus lactis* and *L. garvieae* main clusters. A preliminary genome analysis of *L. garvieae* indicated a pan-genome constituted of about 4100 genes, which included 1341 core genes and 2760 genes belonging to the dispensable genome. A total of 1491 Clusters of Orthologous Genes (COGs) were found to be specific to the 11 *L. garvieae* genomes, with the genome of the “separated strain” showing the highest presence of unique genes.

## Introduction

Over the last decades, the development of efficient molecular methods has revolutionized the microbiological studies and improved the knowledge about the population structure within a single species. The analysis of polymorphisms in a bacterial population, normally subjected to complex processes of diversification, allows the reconstruction of the evolutionary history of a microbe. Various approaches have been developed to trace the history of several bacterial species, including pathogens or opportunistic pathogens. Multilocus sequence typing (MLST) [1] is currently the most widely employed approach to probe the population biology and to predict ancestral genotypes and patterns of descent within groups of related genotypes [2]–[7]. The recent developments in generating whole genome sequences in a short period of time allow to obtain further knowledge about genetic variability [8]–[12]. Today, with the increasing number of complete genome sequences for single bacterial species, that take into account the variability of the dispensable genome, it is possible to trace evolutionary events that have led to genetic changes and that leave a characteristic fingerprint.

*Lactococcus garvieae* (the elder synonym of *Enterococcus seriolicida*) is known as the causative agent of lactococcosis, a septicemic process, described for the first time at the end of the 50s in Japan, in the intensive production of rainbow trout [13].Since then, *L. garvieae* progressively spread in numerous countries and was identified as responsible for outbreaks of this disease in several fish species [14]–[16]. During the last decades, due to an improvement in molecular methodologies, this microorganism, phenotypically similar to the better known *Lactococcus lactis*, has also been isolated in other animal species, as cows and buffalos with mammary infection, in raw cow milk and in human clinical samples [17]–[20]. Genotypic studies were mainly carried out on fish isolates [21]–[23]. The data obtained allowed the differentiation of *L. garvieae* in relation to the host origin and, within the rainbow trout strains, to their geographical origin. More recently, studies carried out on dairy products obtained from raw milk, suggested another possible ecological niche of origin of *L. garvieae*. In some artisanal dairy products, the dominant microbial population was constituted by this species [24]–[25]. Further comparative studies carried out on different isolates, with the aim to identify a possible differential genetic marker, did not produce relevant results. Genes responsible for the utilization of lactose, initially considered specific for the dairy isolates [26], were also found in few strains coming from other sources [27]. The presence of a capsule, previously identified as the main virulence factor in the fish-borne strains, is characteristic to only a few strains isolated from diseased fish and it was not found in strains from other sources [28]–[31]. The absence of capsule was verified by the genome analysis of 11 strains of *L. garvieae*, which over the past two years have become available in public databases, reflecting the increasing interest in the study of this species: five diseased fish isolates [29], [32]–[34], one human clinical isolate [35], two dairy strains [34], [36], one duck intestine isolate [37] and two meat isolates [38]. Recently, we investigated the genetic heterogeneity of a collection of *L. garvieae* strains originating not only from fish and dairy products, but also from food niches not yet studied for the presence of *L. garvieae*: meat products, vegetables and cereals [39]. This strain collection was subjected to typing studies and to a preliminary Multi Locus Restriction Typing analysis carried out on genes belonging to the core genome of the species. The obtained results revealed the presence of at least two genomic lineages within *L. garvieae* population, not entirely coherent with the ecological niche of origin of these strains.

In the present study, comparison among *L. garvieae* available complete genomes, together with multilocus sequence typing (MLST) experiments, were carried out with the aim to better understand the evolutionary history and the genomic complexity of this emerging zoonotic pathogen.

## Results and Discussion

### Multilocus Sequence Typing (MLST)

Nineteen *Lactococcus garvieae* strains were selected from a larger strain collection previously explored through different genotyping methods [39] and chosen as representative of the isolation niche and of the different individuated genomic lineages (Table 1). They were subjected to a MLST that targeted seven unlinked housekeeping genes, possessing the appropriate levels of sequence diversity and lacking insertions or deletions that could cause changes in length. The MLST scheme developed in this study was designed to be technically robust, generating high amplicon yields for all genotypes, under the same PCR conditions for all seven loci. MLST analysis of the 26 tested strains identified 18 different Sequence Types (STs), highlighting a significant heterogeneity in this strain collection. All loci were polymorphic (Table 1). The number of alleles varied between eight in *gap*C, the most conserved locus, and 14 in *rpo*C, suggesting a different evolution rate for different loci, equally distributed along the genome sequences (the minimum distance among the loci was 18 kb).

**Table 1:** *L. garvieae* strains analyzed and allelic profiles.

Strains	Source	Geographical origin	Year of isolation	Sub-groups	Allele							ST	Clonal Complexes (CCs)
					*als*	*atp*A	*tuf*	*gap*C	*gyr*B	*rpo*C	*gal*P		
DSM20684^T^	Bovine mastitis	UK	1984	B	1	1	1	1	1	1	1	1	Singleton
Smp3	Meat products	Italy	2009	B	2	2	2	2	2	2	2	2	Singleton
Po1	Poultry	Italy	2009	B	3	3	3	2	3	3	3	3	1
Tac2	Turkey	Italy	2009	B	3	3	3	2	3	3	3	3	1
Bov3	Beef	Italy	2009	B	3	3	4	2	3	3	3	4	1
I113	Meat products	Italy	2005	/	4	4	5	3	4	4	4	5	Singleton
Ins1	Salad	Italy	2009	B	5	5	6	2	5	5	5	6	Singleton
Sed2	Celery	Italy	2009	A	6	6	7	2	6	6	6	7	Singleton
Br3	Broccoli	Italy	2009	A	7	6	8	2	7	7	7	8	Singleton
Br4	Broccoli	Italy	2009	A	7	6	8	2	7	7	7	8	Singleton
Far1	Wheat flour	Italy	2006	A	8	7	9	2	8	8	8	9	Singleton
G27	Cow milk	Italy	2003	A	9	7	3	4	7	9	9	10	3
G07	Cow cheese	Italy	2003	A	9	7	3	4	7	9	10	11	3
TB25	Cow cheese	Italy	2001	A	9	7	3	2	7	10	9	12	Singleton
G01	Cow cheese	Italy	2009	A	9	7	3	4	7	9	9	10	3
Lg9	Rainbow trout	Italy	2000	B	10	3	4	2	3	3	3	13	1
Lg19	Rainbow trout	Italy	2000	B	10	3	4	2	3	3	3	13	1
V63	Trout	Italy	2003	A	11	6	10	2	9	11	8	14	Singleton
V79	Trout	Italy	2002	A	11	6	11	5	7	12	11	15	Singleton
8831	Rainbow trout	Spain	/	A	11	6	10	2	9	11	8	14	Singleton
21881	Human blood	Spain	/	A	9	7	3	4	7	9	9	10	3
ATCC49156	Yellowtail	Japan	1974	B	12	8	6	6	10	13	12	16	2
LG2	Yellowtail	Japan	2002	B	12	8	6	7	10	13	12	17	2
UNIUD074	Rainbow trout	Italy	/	B	10	3	4	2	3	3	3	13	1
IPLA31405	Cow milk	Spain	2008	B	3	3	4	2	3	3	3	4	1
DCC43	Mallard duck intestines	/	/	/	13	9	12	8	11	14	13	18	Singleton

ST: Sequence Type.

/: Unknown.

The analysis of allelic profiles highlighted a first relationship among strains. Through the eBURST algorithm that defines Clonal Complexes (CCs) by single-locus variants, we identified three main CCs, in which 50% of all the strains were distributed (Table 1). CC1 included seven strains grouped in ST3, ST4, and ST13 sequencing types. CC2 grouped ST16 and ST17, with representative strains ATCC49156 and LG2 respectively. CC3 included four strains belonging to ST10 and ST11. Therefore, the CCs were not homogeneous with reference to the niche of isolation. The remaining 13 strains represented 11 unique STs, indicating a high genotype frequency.

In order to extend the analysis of the genetic diversity of *L. garvieae,* we calculated the average nucleotide diversity π, considering only one sample from each ST. We also measured the π_MAX_, defined as the number of nucleotide differences per site between the two most divergent sequences within the population. This value in fact is not directly correlated to sampling size but only to the extreme values of sequence divergence [40]. The average nucleotide diversity π of *L. garvieae* generated by the analysis of the concatenated DNA sequences of all loci was 0.0297±0.0068, corresponding to 691 polymorphic sites (Table 2). This π value was significantly higher than π for similar species, like *L. lactis* (π 0.0082±0.0010) [40] that appears monophyletic, suggesting the presence of different genetic lineages. For single loci, π ranges from 0.0074±0.0032 for *gap*C to 0.0663±0.0159 for *gyr*B, and these results were also confirmed by the determination of π_MAX_, supporting the hypothesis of different evolution rate of the considered loci.

**Table 2:** Polymorphism observed in seven housekeeping genes in *L. garvieae.*

Locus	Size (bp)	No. alleles	No. Polymorphic sites	π	π _MAX_	Tajima’s D^a^	Fu & Li’s D^a^	Fu & Li’s F^a^	I_A_^S^
*als*	811	11	110	0.0396±0.0086	0.107	−1.068	−1.288	−1.407	
*atp*A	803	9	92	0.0358±0.0117	0.093	−1.133	−0.984	−1.147	
*tuf*	809	12	37	0.0122±0.0022	0.028	−1.163	−1.521	−1.626	
*gap*C	821	8	23	0.0074±0.0032	0.022	−1.6321^b^	−1.7136^b^	−1.886^b^	
*gyr*B	827	11	164	0.0663±0.0159	0.151	−0.703	−0.887	−0.954	
*rpo*C	830	14	79	0.0271±0.006	0.072	−0.701	−1.067	−1.111	
*gal*P	812	13	186	0.0616±0.0188	0.183	−1.413	−1.736	−1.889	
Conc	5713	18	691	0.0297±0.0068	0.091	/	/	/	0.127^c^
Conc S_A_	5713	8	87	0.0063±0.0007	0.009	/	/	/	0.162^c^
Conc S_B_	5713	8	110	0.0071±0.0008	0.011	/	/	/	0.549^c^

Conc: concatenated sequences of seven loci.

S_A_ = Subgroup A, S_B_ = Subgroup B.

^a^ Statistical significance: Not significant, p>0.10.

^b^ Statistical significance: Not significant, 0.10<p<0.05.

^c^ linkage disequilibrium detected.

/: not determined.

π = defined as the average number of nucleotide differences per site for a group of DNA sequences sampled.

π _MAX** = **_maximal nucleotide diversity, defined as the number of nucleotide differences per site between the two most divergent sequences within the population.

**Figure 1:**
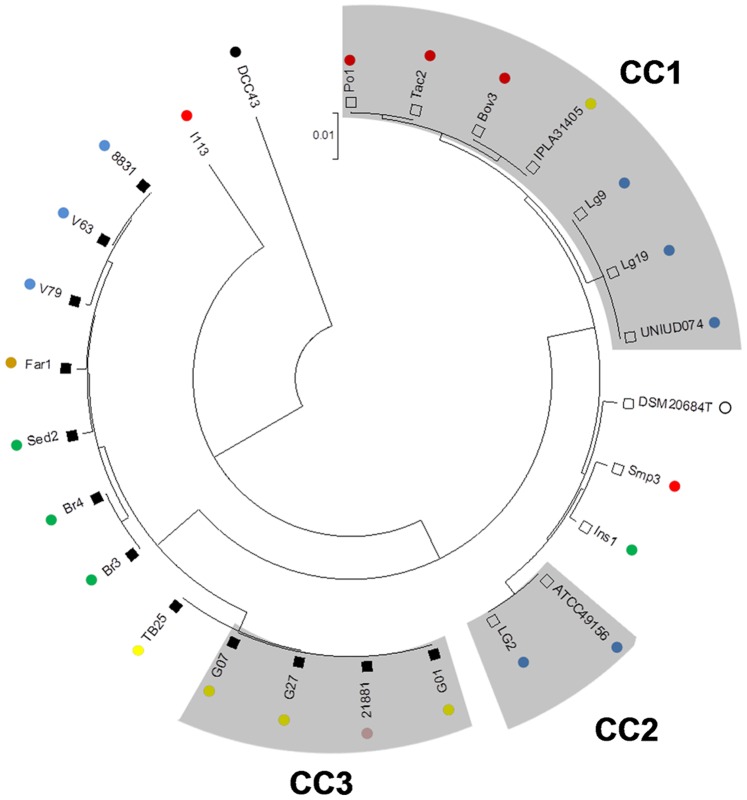
Phylogenetic relationships between *L. garvieae* strains. The unrooted neighbor-joining tree (bootstrap 1000, Kimura 2-parameter model) was constructed from the 5713 bp concatenated DNA sequences of the seven loci (*als*, *atp*A, *tuf*, *gap*C, *gyr*B, *rpo*C and *gal*P) of *L. garvieae*. Open and closed squares correspond to subgroups S_B_ and S_A_, respectively. Strain origin is indicated by color code: green = vegetables, brown = cereals, red = meat, yellow = dairy, blue = fish, pink = human, black = animal intestine, white = mastitic cow. Grey shadows represent CCs.

The phylogeny of the 26 *L. garvieae* strains was analyzed by constructing a neighbor-joining tree from the 5713 bp concatenated sequence of the seven loci (Figure 1). The tree revealed the presence of two main subgroups, as highlighted in our previous work [39]. Subgroup S_A_ consisted of strains included in CC1 and CC2 and three strains with the unique STs. Subgroup S_B_ included strains of CC3 and eight strains with six different STs. Moreover, in this analysis we found that strain I113 and, particularly, strain DCC43 were the most different among all studied isolates, and clustered in independent branches. Strain DCC43 also showed the highest proportion of unique Single Nucleotide Polymorphisms (SNPs) (data not shown). The phylogenetic tree was compared to the topologies of the seven trees constructed for each locus (data not shown). The trees obtained from the analysis of each locus were very similar to the one obtained from the analysis of the concatenated sequence of all loci.

**Figure 2:**
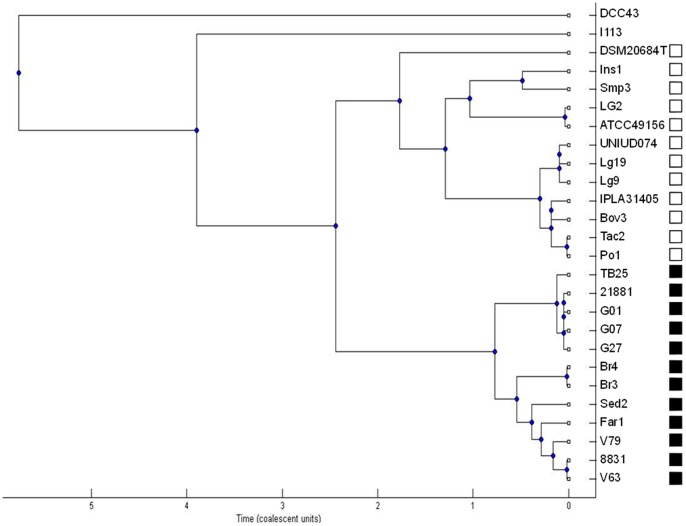
Major rule consensus tree based on Clonal Frame analysis of concatenated sequences of all loci, for the total population. The X-axis represents the estimated time to the most recent common ancestor of *L. garvieae*. Open and closed squares correspond to subgroups S_B_ and S_A_, respectively.

After sequence alignment within the subgroups, the number of polymorphisms and genetic diversity within each subpopulation were reduced (Table 2). This suggests low genetic exchange between these *L. garvieae* subgroups. Moreover, the presence of strains I113 and DCC43, which were not included into any subgroup, significantly influenced the mean genetic diversity of the total population. The Clonal Frame analysis suggests that the two main subgroups appeared at approximately the same time, while ungrouped strains seem to represent the ancestors from which S_A_ and S_B_ originated (Figure 2).

**Figure 3:**
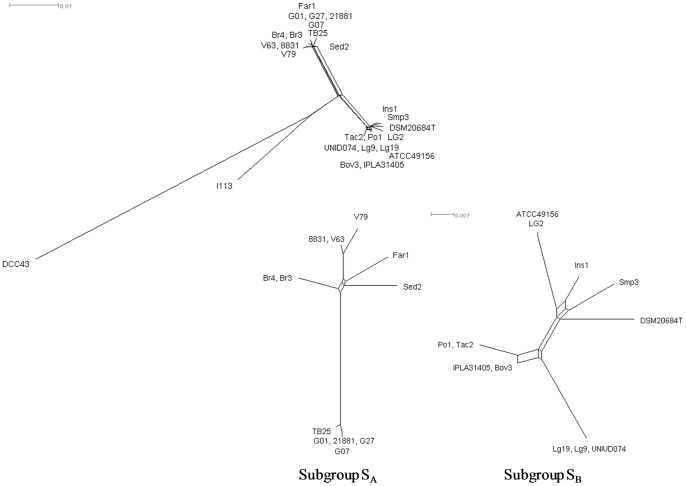
Splits decomposition analysis of *L. garvieae* population and subgroups. Parallelograms identify interconnected network of phylogenetic relationships between strains.

The r/m ratio (ratio of probabilities that a given site is altered through recombination and mutation) was calculated for the entire population and for the two main subgroups, to evaluate whether the high genotypic diversity could be due to recombination events. The r/m was 0.978 for the total population, 0.925 for S_A_ and 1.203 for S_B_. These data probably indicate distinct inclinations and adaptive abilities to environments of the two subgroups: S_B_ seems to respond to selective pressure increasing the recombination rate. It is interesting to note that the recombination events in S_B_ did not seem to contribute to nucleotide diversity (π for S_A_ and S_B_ are similar): recombination among members of the same subgroup did not introduce significative polymorphisms that affect nucleotide diversity. Recombination events in *L. garvieae* population were also investigated using the SplitsTree program, with the split decomposition methods on the concatenated sequence of the total population and for subgroups (Figure 3). Interconnected network of phylogenetic relationships, resembling a parallelogram in shape, was observed. Also in this case, for members of the subgroup S_B_, a major recombinational effect could be highlighted. The tree revealed four major branches: two corresponding to subgroups S_A_ and S_B_ and two longer branches, one harboring I113 strain and the other DCC43 strain. The same analysis was also performed using phylogenetically related *L. lactis* subsp. *lactis* IL1403 (accession number AE005176) and *L. lactis* subsp. *cremoris* MG1363 (AM406671) [29]. The split graph showed the same subdivision of *L. garvieae* population, with the strain DCC43 interconnected with *L. lactis* species by a recombinational event (Figure S1).

Tajima’s D, Fu & Li’s D and F tests of neutrality were used to identify the evolution model of each target gene. All three tests gave values that did not significantly deviate from 0 (p>0.10; for *gap*C locus, 0.10<p<0.05; Table 2), indicating that the seven loci evolved by random genetic drift. The intergenic recombination was calculated by estimating the linkage disequilibrium between loci, using the standardized index of association statistic, I_A_^S^. Only one sample from every ST was analyzed, to avoid introduction of linkage disequilibrium by sampling bias. Significant linkage disequilibrium was detected considering either the 18 STs of the collection (see Table 2), or the two subgroups S_A_ and S_B_. I_A_^S^ was not significantly different from 0, even if subgroup S_B_ showed a higher value, suggesting that the recombination in this cluster has experienced a recent expansion of the population size.

The sequence comparison of 16S rRNA gene, the slowest evolving molecule among housekeeping genes (Figure S2), showed a SNP in the position 203 (V2 region of *Escherichia coli*) [41], distinguishing members of the two subgroups. The strain DCC43 did not belong to none of these groups. Comparison of the 16S rRNA gene showed seven SNPs in other variable regions: two of them, in position 91 and 472, common to *L. lactis*. Thus, even if the strains are closely related in respect to the 16S rRNA gene sequence homology, they are not clustered together (Figure S2), reflecting the subdivision obtained analyzing the other genes.

### Genome Comparison

General features of the *Lactococcus garvieae* genomes are reported in Table 3, in comparison with general genome features of other *Lactococcus* species. For *L. garvieae*, individual genomes varied in size from 1.95 Mb to 2.24 Mb and contained 1778–2227 protein-coding genes. The results include genes that may belong to plasmids or phages [36], [42]. Overall, the genomic variations in size and the number of the protein-coding genes were <15% and <21% between any two strains, respectively. In comparison to other *Lactococcus* species, *L. garvieae* possesses a smaller genome and a smaller number of protein-coding genes. A higher GC content, ranging from 37.70 to 38.80%, was also observed in *L. garvieae*.

**Table 3:** General genome features of the *L. garvieae* strains, in comparison with genome features of *L. lactis* and *L. raffinolactis* strains.

Lactococcus Species	Strain	Genome Size (bp)	GC content(%)	GenomeStatus	No ofprotein-coding genes	Average lengthof protein-coding genes	Protein-coding genesregion %	Intergenicregion %	Accession Number
L. garvieae	8831	2,085,932	38.0	draft	2016	919	88.9	11.1	NZ_AFCD00000000
	21881	2,164,301	37.9	draft	2216	858	87.9	12.1	NZ_AFCC00000000
	ATCC 49156	1,950,135	38.8	complete	1947	874	87.2	12.8	NC_015930
	DCC43	2,244,387	37.7	draft	2227	882	87.6	12.4	NZ_AMQS00000000
	I113	2,178,733	37.9	draft	2159	893	88.5	11.5	NZ_AMFD00000000
	IPLA 31405	2,052,312	38.5	draft	1778	939	81.4	18.6	NZ_AKFO00000000
	LG2	1,963,964	38.8	complete	1968	871	87.3	12.7	NC_017490
	LG9	2,087,705	38.5	draft	2092	881	88.2	11.8	NZ_AGQY00000000
	Tac2	2,242,863	38.2	draft	2210	896	88.3	11.7	NZ_AMFE00000000
	TB25	2,014,328	38.1	draft	2024	879	88.3	11.7	NZ_AGQX00000000
	UNIUD074	2,171,472	38.7	draft	2146	871	86	14	NZ_AFHF00000000
L. lactis subsp. cremoris	A76	2,452,616	35.9	complete	2643	790	85.2	14.8	NC_017492
	MG1363	2,529,478	35.7	complete	2434	856	82.3	17.7	NC_009004
	NZ9000	2,530,294	35.7	complete	2510	837	83	17	NC_017949
L. lactis subsp. lactis	Il1403	2,365,589	35.3	complete	2266	883	84.6	15.4	NC_002662
	KF147	2,598,144	34.9	complete	2444	899	84.6	15.4	NC_013656
L. raffinolactis	4877	2,280,761	38.6	draft	2359	803	83.1	16.9	CALL01000000

**Figure 4:**
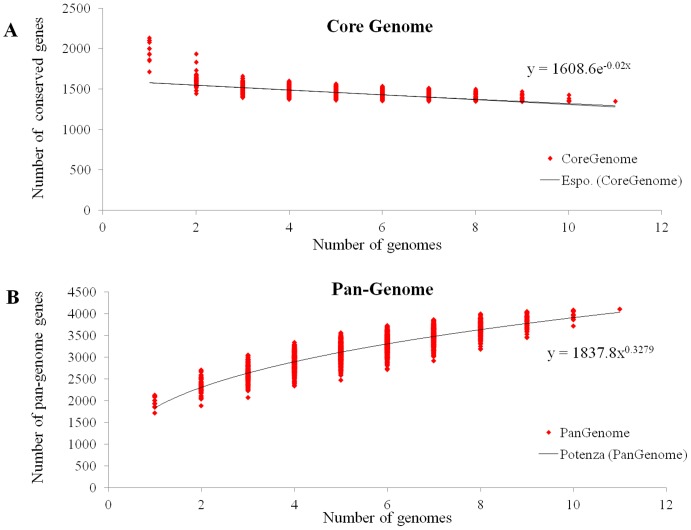
Pan-genome prediction. The distribution of the number of core COGs (A) and total pan-genome COGs (B) found upon sequential addition of *n* genomes. In panel A, an exponential regression to core genome data is shown as a solid curve. In panel B, power law fit to the pan-genome size is shown as solid curve.

To estimate the number of genes present in each *L. garvieae* strain, a pan-genome profile (a full complement of genes in a species [43]–[44]) and a core genome profile (the orthologous genes, OGs, that are conserved in all strains of the species) were built using all possible BLAST combinations for each sequentially added genome. We identified a total of about 4100 OGs. Figure 4 shows the predicted pan-genome size as a function of the number of genomes sequenced. It appears that the pan-genome size is leveling off (at about 4000–4100 genes), as every extra genome adds less new genes. Figure 4 displays the decrease of the core genome as more genome sequences are added. It reaches a minimum of about 1300 genes. In addition, we identified a dispensable genome (present in some but not all 11 strains) of *L. garvieae* consisting of about 2760 genes.

The protein coding sequences of the core were used to construct a phylogenetic tree (Figure S3), which displays the evolutionary development of the *L. garvieae* strains. The tree branching is highly similar to MLST tree generated from the seven housekeeping genes used previously, which highlighted the presence of two clusters encompassing the majority of the *L. garvieae* strains, consisting in subgroups S_A_ and S_B_. Remarkably, *L. garvieae* DCC43 showed a significant genetic distance from the two main subgroups.

In addition, we constructed the evolutionary relatedness between all the *L. garvieae* strains, using a matrix based on the presence/absence of OGs (Figure S4). Although this phylogenomic tree based on the matrix of the total gene presence/absence is different from the phylogenetic tree based on the core genes, the clustering of the strains largely reflects their phylogenetic relatedness.

**Figure 5:**
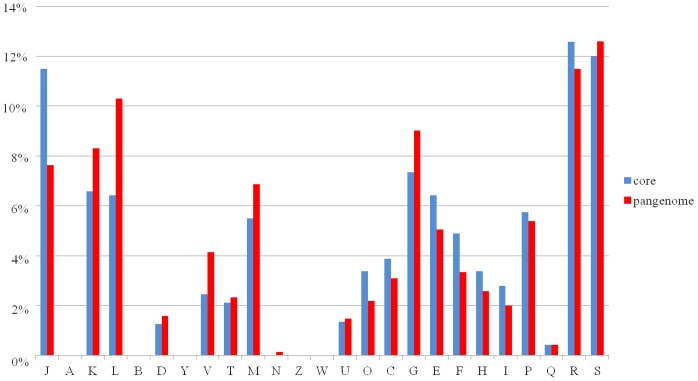
COG families of *L. garvieae*. Bar chart showing a representation of COG families annotation of core COGs and whole pan-genome COGs. Each COG family is identified by a one-letter abbreviation: A, RNA processing and modification; B, chromatin structure and dynamics; C, energy production and conversion; D, cell cycle control and mitosis; E, amino acid metabolism and transport; F, nucleotide metabolism and transport; G, carbohydrate metabolism and transport; H, coenzyme metabolism; I, lipid metabolism; J, translation; K, transcription; L, replication and repair; M, cell wall/membrane/envelope biogenesis; N, cell motility; O, post-translational modification, protein turnover, and chaperone functions; P, inorganic ion transport and metabolism; Q, secondary structure; T, signal transduction; U, intracellular trafficking and secretion; Y, nuclear structure; V, defense mechanisms; Z, cytoskeleton; W, extracellular structures; R, general functional prediction only; S, function unknown.

Figure 5 shows the functional classification of the core and pan-genome genes based on COG analyses. The majority of genes of the core genome belonged to the group of housekeeping functions, as well as other interesting functions, such as metabolism and transport of carbohydrates (G), amino acid metabolism and transport (E), which may suggest that glycans and amino acids shaped the genome of *L. garvieae* taxon. A gene fraction, that appeared enlarged in the dispensable genome, corresponds to defense mechanisms (V) and DNA replication and repair (L). As common in most bacteria, about 25% of the shared genes fall into the class of hypothetical proteins and proteins with unknown function [43].

**Figure 6:**
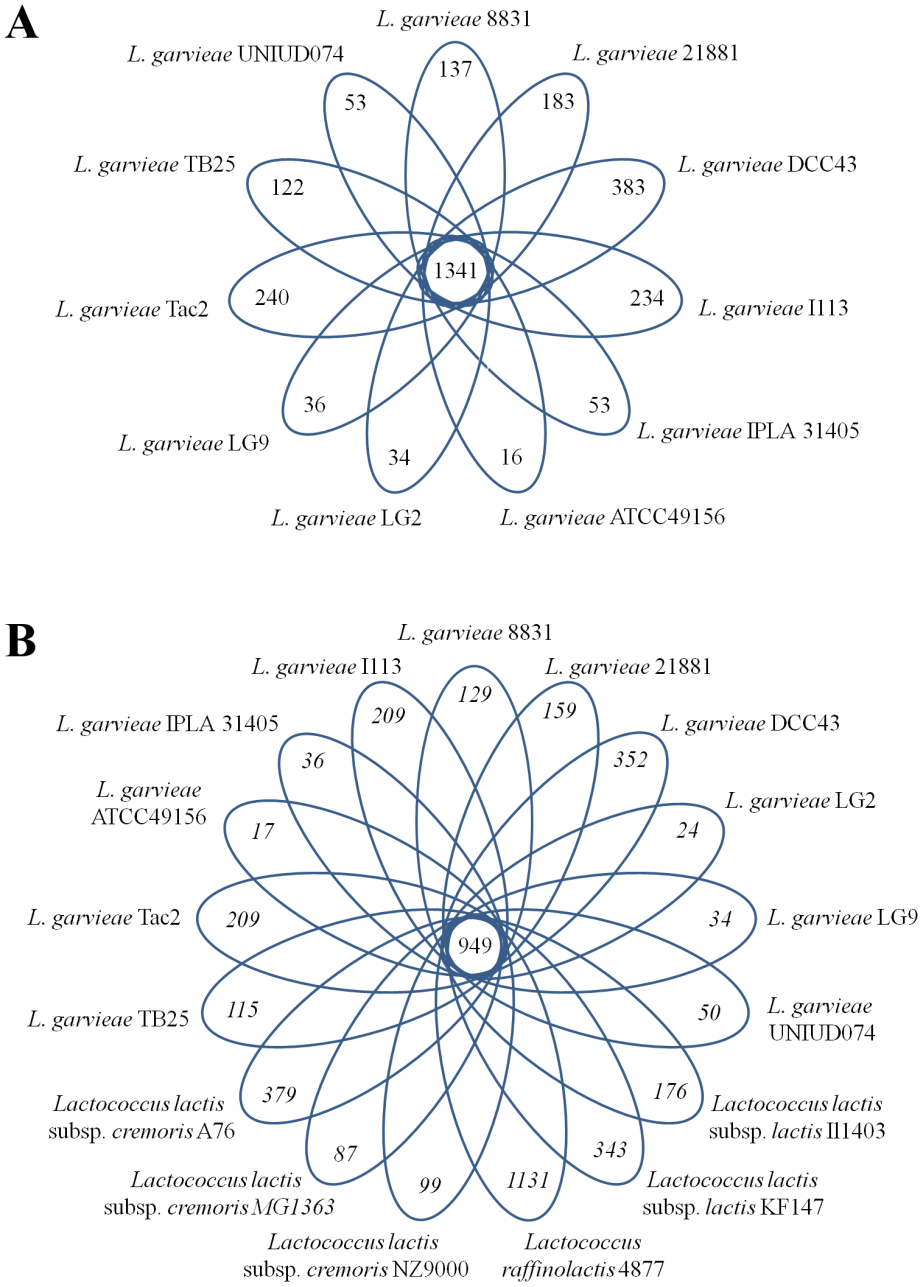
Genomic diversity of the *Lactococcus* species. Venn diagram of core COGs (Clusters of Orthologous Genes) shared between all the strain analyzed and COGs unique to each single strain.

By using the computational procedure described above, we constructed *L. garvieae*- and *Lactococcus*-specific clusters of orthologous genes (LgCOGs and LCOGs, respectively) from the proteins encoded in the genome of the 11 sequenced *L. garvieae*, three *L. lactis* subsp. *cremoris*, two *L. lactis* subsp. *lactis* and one *L. raffinolactis* (Table 3). A total of 1491 LgCOGs were found to be specific to the 11 *L. garvieae* genomes, with *L. garvieae* DCC43 genome showing the highest presence of unique genes (383), representing 25% of the total specific LgCOGs (Figure 6 A). About 70% of the total core genes were also conserved in the six sequenced *Lactococcus* genomes, suggesting that these genes may constitute the core genome of lactococci, likely inherited from a common ancestor (Figure 6 B).

## Conclusions

The lack of knowledge about ecological and functional role of *Lactococcus garvieae* in niches other than fish sector, makes this emerging pathogen attractive to examine in its evolutionary history and in its global complexity. Thus, selected *L. garvieae* strains of our collection coming from various food sources, as well as seven *L. garvieae* genomes of clinical and animal isolates available in databases, were characterized through MLST and whole-genome comparison analyses.

The MLST identified two branches containing the majority of the strains, grouped into two main subgroups, and other two bearing the single strain each. The obtained phylogenetic tree including strains of *L. lactis* subsp. *lactis* and *L. lactis* subsp. *cremoris*, indicates that *L. garvieae* “separated strains” (I113 and DCC43) appeared first and may be considered the evolutionary missing link between *L. lactis* and *L. garvieae.* It is plausible to assume that the strains belonging to the main subgroups could have emerged more recently. Our study also provides a first insight in the core and pan-genome of *L. garvieae*. The core genome consists of 1341 OGs, the dispensable gene pool is estimated to be about 2760 OGs. This accessory genome represents a large proportion of the total genes present within the *L. garvieae* genome, and could suggest the cosmopolitan lifestyle of *L. garvieae* species. Moreover, many genes were found to be specific to the 11 *L. garvieae* genomes, with DCC43 genome showing the higher portion of unique genes.

In accordance to the genetic phylogeny, the comparison of 11 complete genomes of *L. garvieae* highlighted the majority of *L. garvieae* strains to belong to two major subgroups. The obtained consensus tree also suggests the strain DCC43 as the most ancestral lineage of the *L. garvieae* species, when rooted with *L. lactis* sequences. As proposed by Gabrielsen et al. [37], this evolutionary intermediate could represent a novel *L. garvieae* sub-species.

## Materials and Methods

### *Lactococcus Garvieae* Strains

*Lactococcus garvieae* strains tested comprise four strains isolated from diseased fish (Lg9; Lg19; V63, V79), four strains isolated from dairy products (G27, G07, TB25, G01), five strains isolated from meat and meat products (Smp3, Po1, Tac2, Bov3, I113), four from vegetables (Ins1, Sed2, Br3, Br4), one from cereals (Far1) and the type strain of the species DSM20684^T^. For four of these strains, the whole genome sequence was previously obtained (TB25 - accession number NZ_AGQX00000000; Lg9 - NZ_AGQY00000000; I113 - NZ_AMFD00000000; Tac2 - NZ_AMFE00000000) [34], [38]. The strains were grown in M17 broth (Difco, Detroit, USA) supplemented with 10 g L^−1^ glucose (M17-G) at 37°C for 24 h. Stock cultures were maintained at −80°C in M17-G with 15% glycerol.

### DNA Extraction and 16S rRNA Sequencing

DNA was extracted as previously described [39], starting from 100 µL of M17-G broth culture. The concentration and purity of the DNAs were determined with a UV-Vis spectrophotometer (SmartSpecTM Plus, Biorad, Milan, Italy). 16S rRNA amplifications were performed as previously reported [24]. Nucleo Spin Extract II (Macherey-Nagel GmbH, Düren, Germany) was used to purify PCR products that were sequenced using the dideoxy chain-termination principle [45], employing ABI Prism Big Dye Terminator Kit (Applied Biosystems, Foster City, CA). The reaction products were analyzed with the ABI PrismTM310 DNA Sequencer. The database searches were performed by using the basic local alignment tool (BLAST) programs [46] from the National Center for Biotechnology Information website. The phylogenetic tree was constructed using the UPGMA method [47].

### Multi Locus Sequence Typing (MLST)

*Lactococcus garvieae* strains were sequence typed using seven housekeeping genes (*als*, *atp*A, *tuf*, *gap*C, *gyr*B, *rpo*C, and *gal*P). The oligonucleotide primers, designed to conserved regions of the selected genes, conditions used and their amplification products are listed in Table S1, with relevant references. Amplicons were gel purified, sequenced and analyzed as reported in the previous section.

Forward and reverse DNA sequences obtained from PCR amplification were trimmed and studied in comparison with sequences from *L. garvieae* genomes deposited in database (strain 8831 - accession number NZ_AFCD00000000; 21881 - NZ_AFCF00000000; ATCC 49156 - NC_015930; LG2 - NC_017490; UNIUD074 - NZ_AFHF0000000; IPLA 31405 - NZ_AKFO00000000; DCC43 - NZ_AMQS00000000).Selecting the most polymorphic regions of 800–850 bp, these were analyzed using MEGA v5 [48]. Isolate dataset creation and allele assignation was done using PubMLST.org web tools (http://pubmlst.org/analysis/). Each unique allelic profile, as defined by the allele numbers of the seven loci, was assigned a Sequence Type (ST) number. The same ST number was used for more than one strain if they shared the same allelic profile. The number of segregating or polymorphic site (S), nucleotide diversity (π), Tajima’s D, Fu & Li’s D and F were calculated using DnaSP v5.10 [49]. π_MAX_ values were extracted from the squared similarity matrix calculated with DNADIST program (D option set to “similarity table”) in the PHYLIP v.3.69 package [50]. For phylogenetic analysis, concatenated sequences were aligned and analyzed with MEGA v5. Genetic distances were computed by the Kimura two-parameter model, and the phylogenetic tree was constructed using the neighbor-joining method. Strains relationships were analyzed using eBURST [51] to identify potential Clonal Complexes (CCs), with the default stringent (conservative) definition. To investigate the population structure, the Clonal Frame method was used [52]. The recombination to mutation ratio (r/m) was calculated as reported by Vos and Didelot [52]. For each dataset, two runs of the Clonal Frame MCMC were performed each consisting of 200,000 iterations. The first half of the chains was discarded, and the second half was sampled every hundred iterations. Split decomposition trees were constructed with 1000 bootstraps replicates based on parsimony splits as implemented in SplitsTree v4.1 [53]. The standardized Index of Association (I_A_^S^) was calculated with LIAN 3.5 (http://guanine.evolbio.mpg.de/cgi-bin/lian/lian.cgi.pl) [54], using a Monte Carlo randomization test with 1000 resamplings.

### Genome Analysis and Comparison

Each predicted proteome of the analyzed strains (Table 3), was searched for orthologues against the total proteome, where orthology between two proteins was defined as the best bidirectional FASTA hits [55]. Identification of orthologues, paralogues, and unique genes was performed following a preliminary step consisting of the comparison of each protein against all other proteins using BLAST analysis [46] (cutoff: E value of 1×10^−4^ and 40% identity over at least 50% of both protein sequences), and then all proteins were clustered into COGs (Clusters of Orthologous Genes) using MCL (graph theory-based Markov clustering algorithm) [56].

Following this, the unique COGs where classified by selecting the clusters with members from only one of the *Lactococcus* genomes analyzed. COGs shared between all genomes, named core COGs, were defined by selecting the clusters that contained at least one single protein member for each genome. COGs attribution to a specific COG family was made by BLASTp search against the COGs database (http://www.ncbi.nlm.nih.gov/COG/).

In order to provide a highly reliable evolutionary reconstruction, a concatenated protein sequence that includes the product of each core gene from every genome was used to build a *Lactococcus* supertree. Alignment was done using CLUSTAL OMEGA [57], and phylogenetic trees were constructed using the Neighbor joining in PhyML [58]. The supertree was visualized using FigTree (http://tree.bio.ed.ac.uk/software/figtree/).

For all genomes used in this study, a pan-genome calculation was performed using the PGAP pipeline [59]; the ORF content of each genome was organized in functional gene clusters using the gene family (GF) method. A pan-genome profile and a core-genome profile were built using all possible BLAST combinations for each genome being sequentially added. Finally, using the pan-genome profile of shared orthologues between the *Lactococcus (garvieae)* genomes, a pan-genome tree was constructed. This tree was visualized using FigTree (http://tree.bio.ed.ac.uk/software/figtree/).

## Supporting Information

Figure S1 Splits decomposition analysis of lactococcal strains. The concatenated sequences of all loci for *L. garvieae* and for the phylogenetically related species *L. lactis* subsp. *lactis* and *L. lactis* subsp. *cremoris* were analyzed using SplitsTree V4.12. a) Overview phylogeny, b) detail of *L. garvieae* population, c) detail of interconnection among between DCC43 and *L. lactis*.
(TIF)

Figure S2 Multiple alignment of polymorphic sites of the *L. garvieae* 16S rRNA gene sequences. SNPs were reported according to *Escherichia coli* numbering of variable regions (V1–V6) of 16S rRNA gene (Baker et al. 2003). In the UPGMA tree, stratification in subgroup is reported.
(TIF)

Figure S3 Genome phylogeny of *L. garvieae*. Phylogenetic supertree based on the aligned sequences of core proteins shared by all the analyzed *Lactococcus* genomes.
(TIF)

Figure S4 Pan-genome phylogenomic tree built on the presence/absence information of each gene of the pan-genome in each *Lactococcus* genome.
(TIF)

Table S1 Primers used for MLST study.
(DOC)
