# Supplementary figures and images for: Lactococcus garvieae: Where Is It From? A First Approach to Explore the Evolutionary History of This Emerging Pathogen
Source: PLoS One. 2013 Dec 31;8(12):e84796. doi: 10.1371/journal.pone.0084796 (PMC3877359; doi:10.1371/journal.pone.0084796)

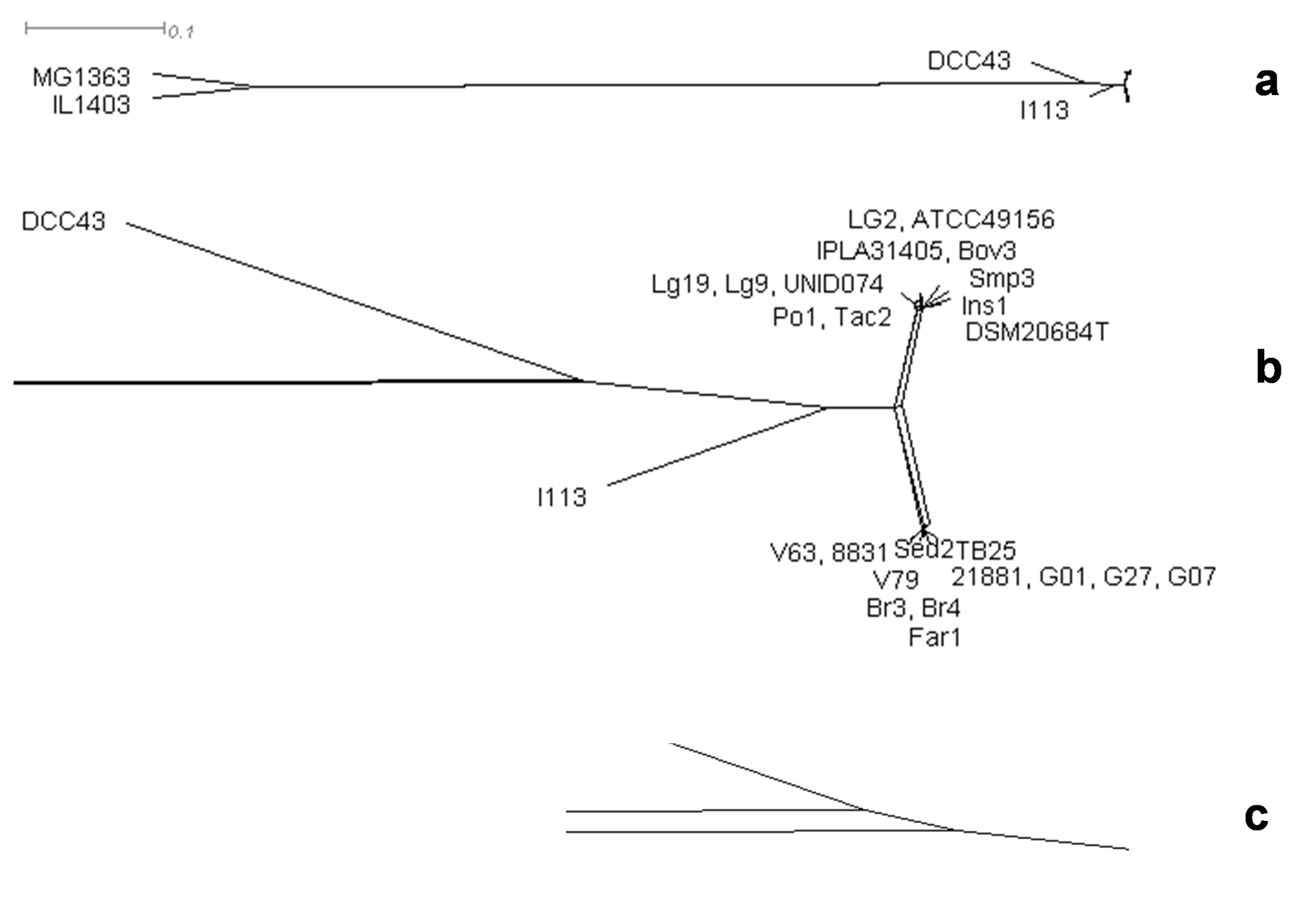

Supplement: Figure S1 — Splits decomposition analysis of lactococcal strains. The concatenated sequences of all loci for L. garvieae and for the phylogenetically related species L. lactis subsp. lactis and L. lactis subsp. cremoris were analyzed using SplitsTree V4.12. a) Overview phylogeny, b) detail of L. garvieae population, c) detail of interconnection among between DCC43 and L. lactis. (TIF) [file pone.0084796.s001.tif]

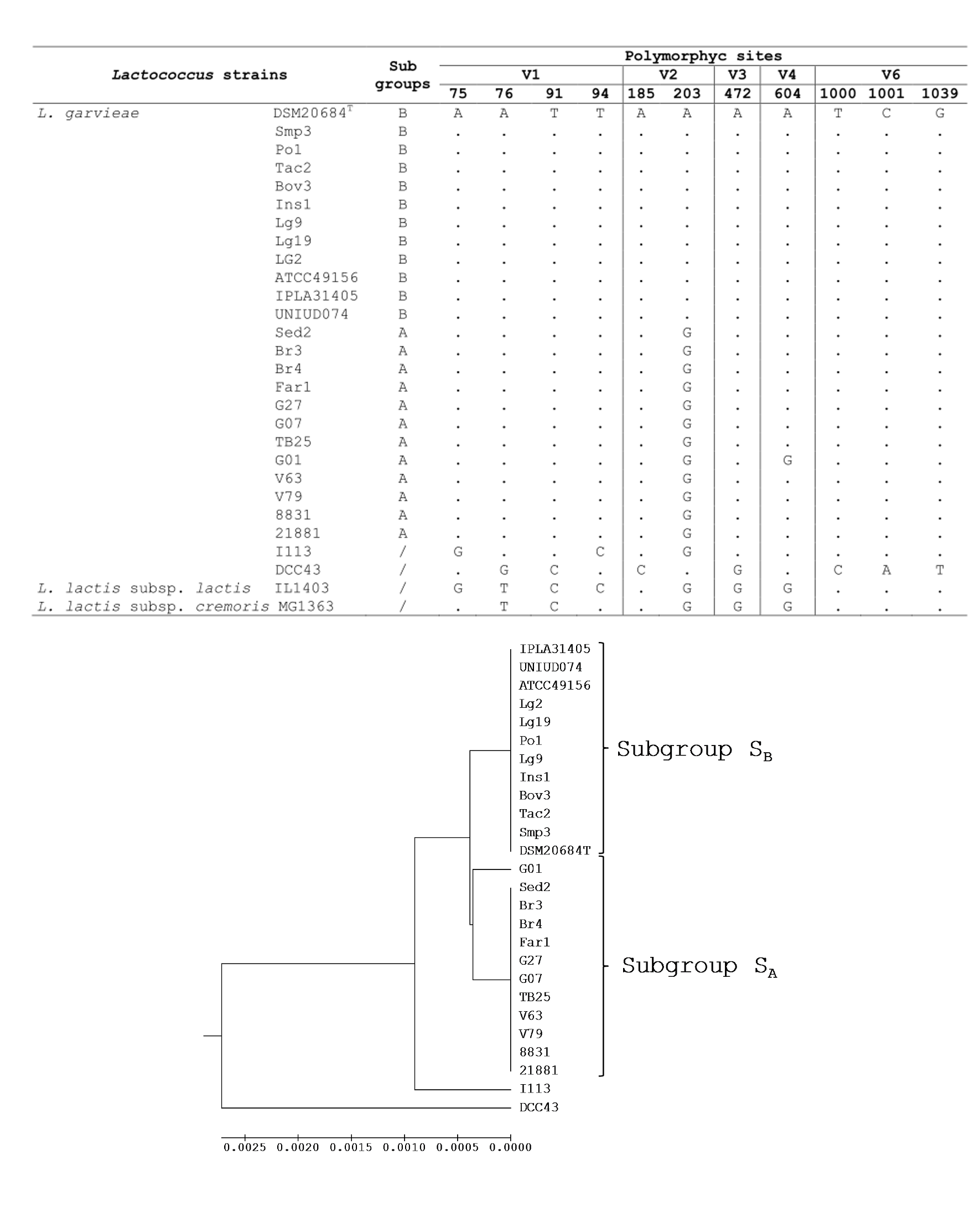

Supplement: Figure S2 — Multiple alignment of polymorphic sites of the L. garvieae 16S rRNA gene sequences. SNPs were reported according to Escherichia coli numbering of variable regions (V1–V6) of 16S rRNA gene (Baker et al. 2003). In the UPGMA tree, stratification in subgroup is reported. (TIF) [file pone.0084796.s002.tif]
