# Supplementary material for: Lactococcus garvieae: Where Is It From? A First Approach to Explore the Evolutionary History of This Emerging Pathogen
Source: PLoS One. 2013 Dec 31;8(12):e84796. doi: 10.1371/journal.pone.0084796 (PMC3877359; doi:10.1371/journal.pone.0084796)
Supplement: Table S1 — Primers used for MLST study. (DOC) [file pone.0084796.s005.doc]

**Table S1.** Primers used for MLST study.

| **Gene /Product** | **Primer pairs (5′-3′)** | **Annealing temperature (°C)** | **Amplicon (bp)** |
| --- | --- | --- | --- |
| *atp*A (α-subunit of ATP synthase)  (Naser et al., 2005) | F: TAYRTYGGKGAYGGDATYGC  R: CCRCGRTTHARYTTHGCYTG | 56 | 1180 |
| *tuf*  (elongation factor EF-Tu)  (Ludwig et al., 1993) | F: ATATGCGGCCGCCATYGGHCACGTBGACCA  R: AAAATATGCGGCCGCTCNCCNGGCATNACCAT | 56 | 1080 |
| *als* (α-acetolactate synthase)  (Menéndez et al., 2007) | F: ATTCGGCTCAGACTTAGTTG  R: TTCAGCTGCTTCAACATCAA | 58 | 1076 |
| *gap*C (glyceraldehyde-3-phosphate dehydrogenase) (Ferrario et al., 2012) | F: AAGTTGGTATTAACGGTTTCG  R: AAGTGTACGAACGAGGTTAG | 56 | 974 |
| *gal*P (galactose permease)  (Fortina et al., 2009) | F: TGGGGAAAATTTAAACCTTGG  R: ATCATCAGAACGGCTGGAAG | 58 | 1070 |
| *gyr*B (DNA gyrase β- subunit) | F: CATGCTGGTGGTAAATTTGG ^a^  R: GTCATCCATTTCTCCTAAACC | 58 | 1464 |
| *rpo*C (RNA polymerase β’-subunit) | F: TTGGTCCACAAAAGGACTGG ^a^  R: TCACGTCCTTTTGCTTCCAT | 58 | 1377 |

^a^: this study

**References**

Naser S, Thompson FL, Hoste B, Gevers D, Vandemeulebroecke K, Cleenwerck I, Thompson CC, Vancanneyt M, Swings J (2005) Phylogeny and identification of enterococci by *atpA* gene sequence analysis. J Clin Microbiol 43: 2224-2230.

Ludwig W, Neumaier J, Klugbauer N, Brockmann E, Roller C, Jilg S, Reetz K, Schachtner I, Ludvigsen A, Bachleitner M, Fischer U, Schleifer KH (1993) Phylogenetic relationship of Bacteria based on comparative sequence analysis of elongation factor Tu and ATP-synthase β-subunit genes. Ant Van Leeuwen 64: 285-305.

Menéndez A, Fernández L, Reimundo P & Guijarro JA (2007) Genes required for *Lactococcus garvieae* survival in a fish host. Microbiology 153: 3286-3294.

Ferrario C, Ricci G, Borgo F, Rollando A, Fortina MG (2012) Genetic investigation within *Lactococcus garvieae* revealed two genomic lineages. FEMS Microbiol Lett 332: 153-161.
